# The Trope of Romani Criminality in Spain's Gender Violence Institutions: A Critique of Punitive Feminism

**DOI:** 10.1177/10778012251379437

**Published:** 2025-09-29

**Authors:** Sarah Werner Boada

**Affiliations:** 1 Department of Sociology, 2707 University of Warwick, Coventry, UK

**Keywords:** Romani women, Spain, gender violence, criminal justice, transformative justice

## Abstract

While Spain has been drawing worldwide attention for its feminist mobilizations in the past years, the movement is currently tearing itself apart on whether a punitive response to gender violence aligns with its values. Based on qualitative research in Madrid's gender violence institutions, I discuss the primacy given to prosecution in the implementation of Spain's internationally acclaimed “gender violence law” and how it interacts with the racist trope of “Romani criminality” in practitioners’ discourses. I show that it manifests in the popular culture myth of Romani men hurting women; strong suspicions towards Romani women summoned at court; and a rejection of Romani resolution process mechanisms. I conclude that Romani women's alternative means of finding safety deserve better consideration for their transformative justice potential.

## Introduction

We demand a feminist justice. A restorative justice that believes us, that centres all the needs of survivors. A transformative justice that seeks to educate rather than punish. We are fed up with revictimization during judicial proceedings. We demand a feminist justice against sexist and racist violence.

Leaflet of the *Comisión 8M Madrid* for International Women's Day, 8 March 2023^1^

The year 2023 marked a significant split within the Spanish feminist movement. A major legal reform, publicly dubbed as the “Only yes means yes law,” had just harmonized the length of prison sentences for convictions related to sexual violence, thus effectively reducing or ending the sentences of some currently incarcerated sexual offenders. The *Movimiento Feminista de Madrid* (MFM), along with various other civil society and political actors, expressed their outrage at the release of convicts who, reportedly, had committed crimes against underage girls and elderly women. Beyond the technicality of the new law's application to individual cases, this sparked a deeper debate and an unprecedently public division among feminists on the use of punitive measures to tackle gender violence. In contrast with the MFM's demand to further criminalize gender and sexual violence, the coalition of regional feminist assemblies, *Comisión 8M*, issued a statement acknowledging the diversity of feminisms and the harm caused by criminal justice to historically oppressed groups such as trans people, sex workers, postcolonial migrants, and other racialized minorities. While barely five years earlier, in 2018, Madrid had come into the international spotlight for its historical International Women's Day mobilization gathering hundreds of thousands of people, in 2023, the day was commemorated by two separate feminist marches in opposition to each other.

Celebrated as the year of the great feminist strike, 2018 had also ended with far more cohesive, nationwide feminist mobilizations around the hashtag “#wearealllaura,” after schoolteacher Laura Luelmo had been sequestrated, sexually assaulted, and murdered by her neighbor Bernardo Montoya in Huelva. Montoya, who is of Romani ethnicity, was featured in local and national newspapers sporting the word “Lucifer” on his T-shirt (ECD, 2018; Moguer, 2018) and referred to as “the *Gitano*”^2^ or “belonging to the *Gitano* clan.” A YouTube video of his arrest in December 2018 showed crowds gathering around the accused and chanting: “Shitty *Gitano*! Assassin!.” Noteworthy, he and his twin brother Luciano had already been the target of local protests and “*Gitano* assassins” graffities in the mid-1990s, after being convicted for the murder of two women in Cortegana. This led many to suggest, two decades later, that Montoya should never have been released from prison in the first place.

Whether or not feminist mobilizations around Luelmo's murder endorsed the racialized discourses mobilized in the press and wider society, the way the case was publicly received remains strikingly reminiscent of a centuries-old trope in Spanish popular culture: Romani men and their “clans” murdering women and abandoning their corpses in mountains. The idea that some groups of racialized men could hold backward views and become uncontrollably violent towards women was, interestingly, already analyzed by Angela Y. Davis, a leading figure of the movement for prison abolition in the USA, almost half a century ago. In her 1978 essay “Rape, Racism, and the Capitalist Setting,” Davis claimed that rumors of Black men sexually attacking white women, which she named “the myth of the black rapist,” had routinely been mobilized throughout US history to condone “waves of violence and terror against the black community” (Davis, 1978, p. 41).

Although the essay more explicitly critiqued unaddressed acts of sexual violence against Black women as a tool of capitalist oppression, Davis's writings have been enshrined in the US Black feminist, prison abolitionist scholar-activist tradition that evidences a continuity between the past enslavement of African captives and the contemporary hyper-incarceration of their descendants in the country. Long discredited by the wider public as at best, a utopian vision of justice, and at worst, a dangerous path towards unregulated violence and impunity, abolitionist feminist initiatives supporting alternatives to the criminal justice system have been gaining ground as a legitimate course of action since 2020, when the Black Lives Matter (BLM) movement publicized the use of police force against Black men (Davis et al., 2022).

Meanwhile, undoing the hegemony of criminal justice remains a difficult conversation among feminist advocates on continental Europe, particularly when it comes to gender violence intervention. Critiques of the law-and-order approach to gender violence and the massively privatized and exploitative “Prison Industrial Complex” (e.g., Davis, 2003; Davis & Shaylor, 2001; Harvey, 2005; Hattery & Smith, 2010; Incite! Women of Color against Violence, 2016; Sudbury, 2005) could easily be dismissed as a USA specificity that is irrelevant to European models of governance. In Spain especially, the 2004 Organic Law on Integrated Protection Measures against Gender Violence—here referred to as LOVG—was, similarly to the 2022 “Yes means yes law,” designed as a comprehensive and survivor-centered law, in an endeavor to go beyond criminal sanctions. It was intended to offer inclusive protection to all women experiencing violence from a former or current male partner, regardless of their legal status or ethnic belonging. Yet, the gender and sexual violence cases that have been making headlines in the past decade do demonstrate that criminalization prevails there too—and more recently, that this approach does not make unanimity. One could understandably wonder whether the growing movement against carcerality among Spanish feminist activists has been imported from the USA without accounting for local differences. However, state-sponsored efforts to maintain Romani communities under surveillance, locked up in a variety of facilities or out of national borders in the name of protecting white Spanish women by far predates the beginnings of slavery in the USA. This paper aims to legitimize the debate on the use of punishment to tackle gender violence in Spain by situating it within the 600-year history of anti-Romani governance in the country and the ways in which this historical legacy still affects Romani survivors seeking support.

In the Spanish context, abolitionist feminism is usually understood as a movement advocating for the criminalization of sex work clients. The feminist voices that denounce the policing of minoritized groups in the name of protecting women thus rather refer to themselves as “anti-carceral” (Alonso Merino, 2023) or “anti-punitive” (e.g., Valenzuela Vela and Alcázar Campos for El Salto, 2023). It has been documented that punitive responses to gender violence, albeit articulated around carceral facilities, go far beyond incarceration to incorporate institutions dedicated to emergency protection but also, more broadly, education, health, family policies or other forms of social support (Valenzuela Vela and Alcázar Campos for El Salto, 2023). Some argue that carceral logics have been taking over welfare-based solutions (Lauri et al., 2023), whereas others address social policy areas as complicit rather than in opposition to carcerality (Brockbank, 2023). In the case of the Romani minority, a deep-rooted system of institutional punishment, relying on spatial confinement and housing evictions, recurrent financial sanctions, and overcriminalization, has been crafted and solidified over centuries, exposing Romani women to violence and rendering it virtually impossible for them to resort to state institutions when in need of support. Hence, while still taking on board critiques of carcerality and the expansion of carceral logics into other institutional areas (see Beckett and Murakawa on the “shadow carceral state,” 2012; or de Finney and Mucina on the “transcarceral pipeline,” 2021), I choose to address the gender violence policy standards and practices discussed in this paper as “punitive feminism,” as they rest upon a broader culture of punishment built to alienate socially and racially “deviant” groups.

Spain's criminal justice system is simultaneously one of the most punitive in Europe (González Sánchez, 2021) and marked by centuries of legislation constructing the Romani minority as naturally prone to crime (Motos Pérez, 2009). In this paper, I show that, due to the primacy given to the prosecution pillar in its implementation, the LOVG operates in ways that harm not only Romani men, but also, and perhaps in even greater proportions, Romani women. The LOVG was formulated with the explicit aim to transform society and protect all women from masculine violence within intimate partnership. I discuss the inherent contradiction in resorting to a set of institutions built to maintain white supremacist patriarchy for gender transformative purposes, and the almost inevitability of failed implementation. Drawing from semi-structured interviews and participant observation conducted within gender violence specialized institutions and with Romani women in Madrid between 2015 and 2017, I analyse the “Romani criminal” trope in the discourses of practitioners, where this trope originates, and how it, ironically, turns Romani plaintiffs into suspects.

I first historicize anti-Romani governance in Spain and examine how criminal justice and mass media work together to propagate the myth of Romani men hurting women. I then add a new gendered layer to the Romani criminal trope by pointing at interviewed practitioners’ differential treatment of Romani and non-Romani women who are in intimate partnerships with Romani men. Finally, I expose the dissonance between institutional feminist solutions to gender violence and the traumatic experiences of Romani women with law enforcement. I conclude that, although interviewed practitioners often felt clueless about how to address the needs of Romani survivors, Romani women have in fact been loud and clear about their demand for agency and respect for the resolution process they resort to as a first layer of protection against institutional racism. Unlike the debate on punitive feminism which has recently surfaced in the public sphere, Romani women's uses of Transformative Justice (TJ) mechanisms are far from new and deserve better consideration.

## Methods

The dataset analyzed in this article was collected in Madrid in 2015–2017, a period of intense feminist mobilizations against gender and sexual violence which led up to the internationally mediatized 2018 strike. Protests such as the 7N *Marcha Estatal contra las Violencias Machistas* in November 2015 or the mobilizations following the Manada collective rape in Pamplona in July 2016 gathered impressive crowds and explicitly targeted state institutions for failing to allocate adequate resources to specialized support services and for downplaying cases of gender and sexual violence. However, discords over the uses and abuses of incarceration were neither prominent nor publicized in the way that they now are.

I conducted semi-structured interviews with practitioners across a wide array of institutions involved in gender violence prevention and intervention but, for the purpose of this article, I will solely focus on those employed in gender violence specialized criminal justice institutions, amounting to 14 interviewees in total. Madrid's gender violence protection network is far denser than in other regions of Spain—there are 11 gender violence specialized courts in the city alone. Within the judiciary, I interviewed the coordinator of the General Council of Judicial Power's Observatory for Gender Violence, five judges, one judicial secretary, and four members of psychosocial forensic teams working in gender violence specialized courts. I also interviewed three police officers at the local police Special Unit for Women, the Youth, and the Elderly. I combined this institutional focus with eight months of participant observation with Romani women attending mandatory welfare programs in the southern suburbs of the city. The interviews I conducted with gender violence practitioners were informed by the women's critical perspectives on the gender violence prevention workshops they were required to attend in return for welfare benefits and by their oftentimes traumatic encounters with law enforcement. Furthermore, I conducted semi-structured interviews with 10 Romani mediators, social workers, and activists working in various civil society organizations or independently. The list of all individual interviewees can be found in the Appendix. Eventually, I discussed my preliminary findings with 40 Romani women attending the programs I had been observing, in the format of four group interviews with 10 participants each.

All interviews were recorded, fully transcribed, and translated from Spanish into English. I coded my dataset with NVivo through thematic analysis, searching for patterns of Romani criminal imagery in research participants’ discourses. The themes generated in the analysis were inspired by Angela Davis's concept of the “myth of the black rapist” (1978) which, she then argued, had been historically constructed in discourses on sexual violence in the USA to justify lynchings—and, further on, the hyper-incarceration of Black men in the country. Given the recurrence of the trope of Romani men murdering women in Spanish popular culture, but also the repeated claims made by Romani women participating in my research that law enforcement targeted them more frequently than men, I aimed to further complexify Davis's argument and look at the various layers of the trope of Romani criminality. I thus explored the themes of violent Romani masculinity, villainous Romani femininity, and primitive Romani community justice. While my use of the word “myth” is intended to explicitly reference Davis, it also echoes recent research conducted by Margareta Matache (2026) on the 500-year-long enslavement of Romani people in the Principalities of Wallachia and Moldavia (nowadays Romania) which, she shows, was justified by “mythmaking” depicting Romani people, including women and children, as criminals. I challenge the narratives around Romani criminality mobilized by practitioners by confronting them to the experiences shared by Romani women during my eight months of participation and our four final group interviews.

### The Myth of Violent Romani Masculinity

#### The Origins of Romani Criminality Fantasies

Attempts at dismantling myths of Romani criminality within Spanish society are rather hopeless if we fail to address the six centuries of governance and cultural production which have shaped them. The specific form of racism targeting Romani people, labelled as Antigypsyism (*antigitanismo*) in the Spanish institutional framework (Alliance against Antigypsyism, 2017; Cortés & End, 2019; Cortés, 2021; Cortés et al., 2021; Hancock, 1997), was formally recognized as a form of hate crime in 2021. The year 2025 was, furthermore, declared by the national government as Day of the Romani People to mark the 600th anniversary of the arrival of Romani people on the territory that later would become Spain.^3^ However, those recent acts of institutional recognition remain superficial and largely performative—while little is being done to address and repair the role played by state institutions, law enforcement in particular, in the oppression of the Romani minority. A precursor to scholarly and policy discussions on anti-Romani racism in Spain was Isaac Motos Pérez (2009) who, over a decade prior to the politicization of Antigypsyism in the country, conceptualized “Gypsiness” (*lo gitano*), the idea that Romani people would be naturally prone to criminality, as central to anti-Romani legislation from 1499 onwards. Even though Romani people's first presence on the Iberian Peninsula was traced back to 1425—that is, before the Spanish nation-state was even built—and they reportedly had arrived carrying letters of protection issued by Pope Martin V to begin a pilgrimage towards Santiago de Compostela (Motos Pérez, 2009), they were rapidly treated as enemies of the Catholic Castilian regime. Uncoincidentally co-occurring with the first European colonial enterprises in the Americas and the expulsion and forced conversion of Jews and Muslims, the royal decree of 1499, issued by the “Catholic Monarchs” Ferdinand II of Aragon and Isabella I of Castile, announced that Romani people would be whipped, mutilated, banned from the country, or enslaved, if they failed to become sedentary (Courthiade, 2019; Motos Pérez, 2009). The decree qualified the Romani lifestyle as “disorderly” and as setting a poor example for the rest of the population. Incarceration, forced labor, and enslavement for life were recurrent sanctions inflicted on Romani people throughout centuries on the ground that they were dangerously wandering. A further decree issued in 1633 declared: “they do not proceed from any origin or any nation, they are merely lazy people who have adopted this way of life in order to be able to carry out their misdeeds” (Motos Pérez & Caro Maya, 2015). From the late seventeenth century onward, they were required to register as “new Castilians” and to settle in specific localities where they would remain under surveillance (Motos Pérez & Caro Maya, 2015). Their right of asylum was withdrawn in 1737 through a Concordat with the Holy See and, in 1749, an estimated 12,000 were arrested (Courthiade, 2019) and for the most part only set free in 1783 (Motos Pérez & Caro Maya, 2015). Importantly, the 1783 decree issued by King Charles III which put an end to their reclusion still qualified them as dangerous before even committing any deed—and thus, according to Motos Pérez (2009), represented a turning point in Spanish criminal law. The explicit reference to Romani people's predisposition for crime was to remain enshrined in the legal framework up until 1978.

Conveyed by countless works of fiction considered national landmarks, from Miguel de Cervantes’ *La gitanilla* (1613) to Emilia Pardo Bazan's *La novela de* *Raimundo* (1898), the myth of Romani criminality however never quite left the public imaginary. Ismael Cortés (2020, 2021) argues that media tales of Romani violence—alleged rifts between “clans” “within the ghetto” and extreme displays of violence against innocent white people “outside the ghetto”—are to this day still mobilized to instil fear of a “*Gitano* threat” within society (Cortés, 2021, p. 97). What is often overlooked by critical commentators, and yet central to this imagery, is the fact that fantasies of Romani criminality overwhelmingly refer to femicides—gender-motivated murders of women perpetrated by men. While Cervantes and Pardo Bazan described Romani men as “[murdering and burying their wives and girlfriends] in the mountains and deserts as if they were harmful animals” (Cervantes 1613 as in Pardo Bazan, 1898, p. 6), contemporary media representations portray them as hypermasculine and fueled by anger, either engaging in revenge murders within their communities, or seeking to harm innocent white women, as in the case of Bernardo Montoya.

#### The 2004 Gender Violence Law

Graphic depictions of violence and stereotypical portrayals of perpetrators and victims in the media are an efficient means to build public support for punitive measures. Brutal rapes and femicides often become the catalyst for the adoption of legal reforms criminalizing sexual and intimate partner violence, a phenomenon which Kristin Bumiller refers to as “expressive justice” (2008, p. 26). In Spain, too, the laws and policies which laid the foundations for the 2004 LOVG were adopted following a series of highly mediatized crimes throughout the late 1990s. The National Action Plan against Domestic Violence 1998–2000 was approved merely a few months after the murder of Ana Orantes, who was immolated by her ex-husband after appearing on television. It justified introducing more severe criminal sanctions by arguing that “[domestic violence had] become a threat to society and an essential attack to democracy” (p. 1, quoted and translated in Roggeband, 2012, p. 794). Further sanctions were introduced into the Criminal Code with the Organic Laws 11/1999 and 14/1999 (Roggeband, 2012, p. 794).

Admittedly, this punitive turn was received with scepticism by many feminist advocates. As in other regional contexts, feminist initiatives against gender violence in Spain had begun organizing outside the state in the 1970s—but also explicitly against it, as Francisco Franco's authoritarian regime confined women to patriarchal gender roles and relied on state repression and incarceration into various forms of facilities to punish social deviance. The legal codification of gender violence and, more specifically, the use of punishment to protect women has been described by some scholars as antithetical to feminist values (Laurenzo Capello, 2005; Restrepo Rodríguez & Francés Lecumberri, 2016).

After several years of lobbying, the LOVG was drafted as an attempt to center state intervention on survivors’ needs and social transformation. The law groundbreakingly includes emergency and long-term protection measures, including free legal aid and social assistance, the eviction of perpetrators from the home in case of shared household, unemployment and social reintegration benefits, work leaves, geographic mobility support, as well as the free issuance or renewal of temporary residence and work permits for undocumented plaintiffs. Its multi-sectoral, comprehensive approach provides for primary and secondary prevention measures in partnership with the education system and the media, but also a connection between criminal and family legal matters through the creation of specialized courts—the so-called Violence against Women Courts (*Juzgados de Violencia sobre la Mujer*),^4^ which have competence for both criminal and civil law. This double competence ensures divorce and child custody decisions are incorporated into the ruling and that plaintiffs avail of the administrative and financial means to live in safety after filing a complaint.

However, what has mostly brought public attention to the LOVG is its definition of gender violence as perpetrated by a man against a woman within intimacy, thereby introducing more severe criminal sanctions for male perpetrators. Following its adoption, law enforcement entities established coordination with gender violence courts through national digital databases, daily communication, and follow-up protocols with victims, in theory even when the latter chose not to file a complaint or when the suspect was not convicted. Yet, while effective coordination is considered an important standard in state response to gender violence, it is striking that, in Madrid at least, most efforts have been concentrated on the prosecution pillar, and coordination between gender violence courts and specialized support services was practically non-existent at the time of my research. None of the specialized judges and court staff members I interviewed knew what happened to victims after judicial proceedings.

Despite feminist endeavors to shift the focus from punishing perpetrators to offering comprehensive support to survivors, the LOVG is mostly known in Spanish society as the introduction of gender-differentiated criminal sanctions and its implementation efforts are evidently channeled into punishing violent masculinity.

#### Over-prosecuted minorities

The rallying of feminist agendas to the modern carceral state not only enters into contradiction with 1970s radical feminist critiques of criminal law and prisons as producers of violent masculinity—it also becomes a powerful strategy to target minorities. Elizabeth Bernstein (2007, 2012) refers to feminist support for increased punishment and incarceration to protect women from violence as “carceral feminism.” Under neoliberal governance, incarceration has skyrocketed as a means to rid society of minoritized groups instead of providing them with equal rights and social support. In the USA, Black men are overrepresented in sexual infraction files (Hoppe, 2016; Ricordeau, 2019) and incarceration and eviction rates of Black and other racialized minority men have been steadily increasing along with the law-and-order approach to tackling sexual and intimate partner violence (Meiners, 2009; Richie, 2000; Ricordeau, 2019).

Although critiques of criminalization of gender violence in Spain tend to overlook race, there are reasons to expect that higher chances of prosecution and incarceration similarly await perpetrators from racialized minority groups. Judicial statistics on gender violence collected annually between 2011 and 2020 arguably show that gender violence-related convictions are significantly lower than for other crimes (Consejo General del Poder Judicial, 2021, p. 30). However, they do also show that non-nationals are three to four times more represented than Spanish nationals among the accused (Consejo General del Poder Judicial, 2021, p. 22). This should hardly come as a surprise, given the history of governance of minorities in Spain. The Spanish criminal justice system is reportedly one of the most punitive in Europe despite a relatively lower criminality rate (Brandariz García, 2015; Díez Ripollés, 2006), with average prison sentences that are twice as long as in other European countries (González Sánchez, 2021). The repression for which Franco's ultra-nationalist dictatorship was known throughout most of the twentieth century has naturally left scars in the country—but in fact, Spain's prison system has become even more punitive since the so-called transition to democracy. While the impressively high prison population rates dropped due to budget cuts in carceral facilities after the 2008 financial crisis (Brandariz García, 2015), state authorities then started to multiply deportations of migrants as well as armed police patrolling to increase surveillance of minorities (Ballesteros Pena, 2020; Brandariz García, 2015; González Sánchez, 2021). Many activists and prison studies scholars have raised concerns over the overcriminalization of migrants and appalling conditions in Foreigner Internment Centres (*Centros de Internamiento de Extranjeros* [CIE]) (González Sánchez, 2021).

As Ignacio González Sánchez affirms (González Sánchez, 2021), there is a daunting lack of data on the prosecution and incarceration of Romani people in the country, both because public authorities are not permitted to collect ethnically disaggregated data and because many researchers continue to have anti-Romani prejudices. It is therefore hard to make further claims on the incarceration of Romani men for gender violence crimes. That said, several gender violence specialized practitioners I interviewed in Madrid mobilized similar stereotypes of violent Romani masculinity to the ones one can find in the media. A gender violence court judge (interviewee #3) for instance shared the following anecdote, almost referring to it as entertainment:

Last week […] we had here, so to speak, a spectacle that could have been very serious, because there was an attempt at shooting, there was a *Gitana* woman… I am not in charge of this case […]. She had filed for a protection order, he had been arrested, she came to testify, and his family was there, down on the street. They attacked her. Someone from the family, apparently took a gun out. And shot her. The shot did not come out because the gun jammed. Anyway. It was such a mess in here, seven police cars came, the military police that is usually here to watch detainees came out, and we were here, working, when suddenly I see, we could hear police sirens. […] I see some police officers running out, looking for the weapon… Well. A real show […]. The one with the gun, they didn’t manage to catch him. He escaped. […] She came to testify and, what she said was that the family mediation had not worked out and that […] she had come here convinced that they were going to end up killing her. (Sighs)

Particularly for a minority who, due to a traumatic past, mistrusts law enforcement and criminal justice institutions in general, it is to be expected that cases brought to gender violence courts, whether through third-party reporting, emergency police intervention, or a victim filing a complaint because she fears for her life and her children's, would more likely be cases of extreme physical violence. Yet, perhaps biased by media reports, many practitioners assumed those cases were representative of Romani customs. Similarly, a gender violence court psychologist (interviewee #8) suggested that a Romani woman filing a complaint for gender violence could lead to community revenge murder:

Many experience reporting as betrayal, so they feel guilty, they feel they have to justify their partners’ behaviour, minimise it, negate it […] it is very difficult for them to report! And then another thing is, they are very much threatened! Sometimes of being killed… or that something might happen to their own family… […] So it has a lot to do with the risk they see, the consequences that a separation could lead to! Not only for themselves but also for their own family! Because maybe, he is threatening them, but the *Gitano* family also is threatening!

Once again, a justified fear of retaliation after reporting, which is shared by many gender violence survivors, becomes conflated as a Romani specificity. Reminiscent of the tales told by Cervantes and Pardo Bazan—and other cultural references—several interviewed practitioners (#3, 5, 6, 7, 8, 11, 13, see Appendix) referred to Romani people as “clans,” “collectives,” for whom it is relatively common practice, and expected, to punish their wives in case of deviant behavior (Cervantes Saavedra, 1867). This narrative was especially prominent among interviewees who claimed to espouse a feminist reading of intimate partner violence, including a highly ranked gender violence specialized Magistrate who regretted that “they [were] not civilised like us” (interviewee #11), and the interviewed members of gender violence court psychosocial forensic teams who for example concurred that “*gitano* communities [were] not integrated” (interviewee #8) and had “a patriarchal system that [was] a lot more obvious that in other cultures,” with “barbaric stories” (interviewee #5). This culturalization of gender violence has been critically analyzed by various feminist scholars, including at the EU level (Montoya & Rolandsen Agustin, 2013). What should remain clear is that, consciously or not, the purpose of this culturalist narrative is to lock up—or out (Farris, 2017)—racialized minority men, rather than truly protect women. This becomes visible when interviewees delve further into plaintiffs’ backgrounds and potential defence strategies.

### The Myth of Villainous Romani Femininity

#### Differential treatment of plaintiffs

An important contribution of abolitionist feminist writings on criminal legal responses to gender violence has been to show that survivors themselves are exposed to harm in judicial proceedings when they belong to historically oppressed minorities (Richie, 2012; Vergès, 2020). Drawing on Bernstein's critique of carceral feminism, Françoise Vergès (2020) argues that under the falsely universalist guise of protecting all women from violence, the hegemony given to the prosecution and incarceration route in gender violence legal standards, in practice, throws minority women under the bus to save women from privileged groups only (Bernstein, 2007, p. 137; Vergès, 2020, p. 96). So long as the women who correspond to ideals of victimhood can benefit from coercive measures such as mandatory reporting, mandatory arrest, and increased prison sentences, it is tacitly considered acceptable to expose others to further police surveillance which, in cases of stigmatized and economically dispossessed groups, can have devastating consequences, such as child custody loss, eviction, or incarceration for poverty-related petty crimes. This rapidly became evident in the discourses of some interviewed practitioners.

Indeed, one might have expected that practitioners resorting to the violent Romani masculinity narrative would automatically have constructed the partners of the accused as victims. However, many held double standards when assessing the circumstances which led the plaintiffs to go through judicial proceedings, depending on whether the latter were themselves Romani or not. Hence, three interviewees working within gender violence court psychosocial teams (interviewees #5, 7, 8) expressed sympathy for non-Romani women who, they claimed, had entered violent partnerships with Romani men because they were vulnerable and isolated. One of them (interviewee #7) thus regretted that they were dragged into Romani patriarchal customs because of their relationship:

They are *payas* [non-Romani], but at the end of the day […] the same is required of them as of the *gitanas*, because they are the woman of a *gitano*.

Meanwhile, while observing emergency courts hearings, I witnessed court practitioners either suggesting that Romani plaintiffs were making up testimonies or trivializing their experiences as typical of Romani customs and, therefore, far less harmful for Romani women than for non-Romani women. During one observation, I was told by the team on duty about a young Romani plaintiff who had filed a complaint against her ex-partner but then changed her testimony after being summoned at court. Unwilling to testify but legally obliged to do so, she reportedly declared in front of the judge that her memory had been blurred by alcohol. In a whisper, one of the legal interns present in the room mocked the plaintiff's mother for “smelling” and complained that they had “wasted [everybody's] time.” No consideration was given to this woman's safety, the veracity of the complaint that had brought her to an emergency hearing, or why she might have felt she needed to lie in court. Another plaintiff whose file I was granted access to had similarly changed her story and requested to drop rape charges against her ex-husband, who had broken into her house through a window and assaulted her. The judge in charge of the case (interviewee #3), deeming her account incoherent, laughed in response:

Well, she did have eight children with him, right? It's hard to believe she would be forced to have sex?

Furthermore, in contradiction with the general emphasis on support networks of families and friends being a crucial safety factor for plaintiffs during proceedings, several interviewees considered Romani relatives’ presence in court hearings to be disruptive or, worse yet, an obstruction to justice. A specialized criminal court judge (#10) expressed concerns over the “ton of [Romani] family relatives [showing] up” who, she thought, “may exert pressure on the person testifying.” A gender violence court social worker (#6), supported by a colleague from another psychosocial forensic team (#5), complained that their presence “[affected] the staff in the courts,” and a gender violence court judicial secretary (#10) went as far as saying that judges were too scared to “confront the *gitanos.*” While secondary victimization during proceedings is unfortunately widespread even in specialized courts, the change of tone to refer to Romani women's testimonies and needs was striking.

Even though the practitioners who accepted to be interviewed visibly did so because they were keen to improve support for Romani survivors, their discourses remained tainted with subconscious bias which, once again, must be situated within the anti-Romani public imaginary maintained through legal practice and cultural production since the fifteenth century. Both Patricia Caro Maya (2019) and Pastora Filigrana (2020) have argued that state and church authorities throughout Spanish history specifically targeted Romani women whose self-reliant outdoors activities incarnated the opposite of what was expected of women in a Catholic regime. Romani women were banned from selling in the markets, but also, they were accused of witchcraft, spying, and prostitution, and were detained in the bishopric's houses of correction after the 1749 mass arrest known as the Great Round-Up (Gómez Alfaro, 2010). Most of us will have encountered the figure of the promiscuous, deceitful “*Gitana* woman” in popular songs, tales, or childhood cartoons. The trope of Romani criminality has gendered variations—and it is so deep-rooted that Romani women experiencing similar patterns of abuse to non-Romani women could not possibly be considered as victims in the same terms by practitioners when seeking help.

Worse yet, they may well risk prosecution and incarceration themselves. Albeit counter-intuitive, the criminalization of survivors of gender violence is not unknown of, especially when they belong to historically policed minorities (Richie, 1996, 2012). In Spain, gender violence survivors and non-nationals are overrepresented in prisons for women (Ballesteros Pena, 2020). Although there is no data available, it is perfectly plausible that Romani women who have been incarcerated have also experienced gender violence. Research conducted in 12 prisons across the country in 1999, known as the “Barañí project,” exposed an astounding overrepresentation of Romani women in Spanish prisons: 20 times higher than in wider society (Martín Palomo, 2002). It showed that the vast majority had been directly convicted for long prison sentences, despite being judged for petty crimes like petty robbery and small-scale drug trafficking, and having often perpetrated those crimes for the benefit of their husbands or sons (Martín Palomo, 2002). Barañí research team member Teresa Martín Palomo claims that, because no legal complaint is needed, the criminalization of drug-dealing and drug use opens the door to further discretionary practices at the hands of state authorities (Martín Palomo, 2002). It may easily be mobilized as a ground for detaining victims rather than providing them with support. Yet regardless of whether they had been involved in petty crimes, Romani women described by interviewed practitioners were disproportionately judged responsible for acts of violence perpetrated within their communities—and their caretaker and family pillar roles were especially put into question.

#### Dangerous mothers

Another way the Romani criminality narrative is gendered is the construction of Romani women as irresponsible or, in some instances even, abusive mothers. Not only did several interviewed practitioners doubt Romani women's motives and testimonies, but they also held them responsible for, allegedly, tolerating and reproducing gender violence across generations. Many interviewees (#5, 6, 7, 8, 11, 13) supported the common myth that Romani communities are ruled by so-called patriarchs who decide over the fate of women and girls, and thus attested that Romani women were sucked into patterns of violence from an early age. A police officer, working for the local police Special Unit for Women, the Youth, and the Elderly (interviewee #13), argued that gender violence was so deeply enshrined in the Romani lifestyle for generations that Romani women were doomed to experience it without questioning:

It's true that some of them […] maybe have other ideas, a little more liberal so to speak (laughs) […] but with time, they still end up adopting the same behaviour as their mothers. And in addition, they generally live with their parents-in-law, and of course, you witness it every day from them, at the end of the day you experience it too, you trivialise it! […]

So in the end, […] well, getting rid of this whole mentality, it needs to be changed, but it won’t happen from one day to the next! It takes generations…[…] How do you do it? Tell them ‘it's over now!’… but it's their way of life! They don’t know any other way! […] And you cannot avoid it, because (*speaking on their behalf*) I am learning this from childhood! As I don’t have any alternative because I’ve never seen anything else, because they’ve never shown anything else to me, I understand that it is normal. And that's how it is… one case after another.

It is noteworthy that, rather than focus on intimate partners’ coercive control strategies—as gender violence specialized practitioners should be trained to do—the interviewee claimed that Romani girls, even if they initially had other life plans, started experiencing violence because they modelled their mothers’ behavior. This type of narrative suggests risks of institutional neglect—since it is assumed that things will never change—or, even worse, child custody withdrawals. It is especially surprising that it can be mobilized by practitioners who usually work with a systemic gender reading of violence and have long lobbied to address secondary victimization and child custody loss during judicial proceedings. This indicates that, despite the enormous efforts which feminist advocates have deployed over the years to lobby for a survivor-centered legal framework, few have genuinely considered the perspectives of Romani survivors.

### The Myth of Primitive Community Justice

#### Rethinking Safety

The Spanish state has been applauded multiple times for setting up, from the 1990s onwards, partnerships between regional authorities and local non-governmental organizations to offer empowerment programs for Romani women. The understanding is that attending those programs several hours a week provides women with an escape from their stifling heteropatriarchal household, a space where they can finally breathe, focus on their own well-being, and learn to recognize patterns of gender violence in their homes. For eight months in 2016–2017, I attended the weekly activities of an organization based in the southern outskirts of Madrid, where many Romani people live. Participation in the activities was a non-negotiable condition for Romani women who received welfare benefits to provide for their households. Towards the end of my stay, I conducted four group interviews with program attendees within the organization's facilities to discuss my preliminary research findings. I reported to them the broad themes that had emerged from my interviews with practitioners, and asked them the extent to which those representations matched their personal experiences, with a focus on their perspectives on everyday and emergency safety. Each group interview was one hour long and included ten participants. None were recorded and participation was optional. Upon asking all participants about the places where they felt safest, I was offered a radically different view of safety. The responses were unanimous: home—sometimes broadly defined as including their direct neighborhood and church—was the only place where they did not feel threatened. One participant tellingly fantasized: “I wish I could take my children, stay home, lock the doors and throw out the keys.” Through my regular interactions with program attendees during my eight-month participant observation at the organization, it quickly became clear that the outdoors represented a place of high vulnerability for them. I was struck by their frequent complaints that gender violence and empowerment workshops felt unnecessary and condescending. They also recurringly reported being subjected to humiliating policing at the hands of law enforcement and other institutional actors. Police patrols and private security guards alike arbitrarily put them through ID and permit controls on the streets and the markets, searched them for drugs, and accused them of stealing—regardless of their age and physical condition. During one of the interviews, one of the participants, supported by emphatic nods from other women in the group, informed me that law enforcement targeted Romani women and children far more than men, because women had a “reputation” for stealing and “hiding things under [their] skirts”. While they shared disheartening stories of social services visits turning into police interrogations, they converged in saying that police officers were the worst because, in the words used by one of them, “they allowed themselves to do anything”. More alarming yet was the fact that they felt no one would ever defend them. One participant shrugged in disbelief when I asked about finding support in such situations: “and who is going to help us?”. The general consensus, across all four groups, was that the police was racist and should only be resorted to as a last resort, if at all, when faced with a dangerous situation.

Indeed, law enforcement have been trained and used at various levels of governance, across six centuries, to rid the Spanish nation of unwanted bodies and practices. The ideology propagated by the Catholic Monarchs from the early days of Spanish imperialism is so deeply engrained in the common imaginary that a hospital staff member went so far as to declare to one of the Romani mediators I interviewed (#15): “Such a shame that the Catholic Monarchs are no longer alive in Spain!”. How could one possibly expect Romani women to trust public authorities—let alone police forces? Yet, interviewed gender violence practitioners could not comprehend why Romani people acted so defiant with them. One of the police officers (#13) in fact interpreted this negative attitude as frustration because the police prevented them from exerting illegal activities:

They don’t consider that we do what we do to help them. They don’t. […] They do not get on excessively well with the police to put it that way, no, because they hate police officers… Well, logically you do your job, and maybe… well maybe that's not what they want (*laughs*) you know? And then, well, it's true that maybe, if they sell fruit and don’t have a permit, well… you’re going to take away their fruit and report them… If they do something else and they don’t have the corresponding documentation, they don’t have a licence, you take away their car, you report them… They see it… well, as a threat.

Although Romani group interviewees did indeed describe police intervention as a threat, this police officer merely interpreted Romani people's distrust of the police as a fear that someone could put an end to their undeclared economic activities and other forms of illegalities. She considered that police work was “help” and did not seem to think for a second that it could be perceived as frightening or dangerous. What came out, albeit more cautiously, from my participant observation and group interviews with Romani women, was that if they felt endangered in their homes, their preferred way to exit violence often was not what was offered by the criminal justice system.

From an institutional feminist perspective, resorting to alternative routes seemed unthinkable. All this work to set up a legal framework protecting women from violent male partners and their entourages—yet survivors continued to turn to them instead? Initially contacted to discuss the rapport between Romani women and gender violence specialized institutions, all the gender violence practitioners I spoke to, without exception, interpreted the purpose of my research as finding a way to reach out to women who were lacking information about their rights and about the legal and social support made available by the state. The most highly ranked judges who participated in the research (interviewees #10 and 11) both made a point of highlighting the need for more targeted awareness campaigns and prevention work specifically designed for Romani women, who were “uneducated” (interviewee #11) and lived too far in the margins of society. My interview grid's question about the potential impact of racial stereotypes on institutional practice—which I would systematically ask half-way through the interview—was often received with shrugs or even dismissals, including a defensive response from gender violence court social workers #5 and #6 who retorted back that stereotypes were well-founded.

More specifically, practitioners feared community elders’ resolution processes, developed by Romani people in Spain to cope with a hostile state, as a form of gender violence orchestrated by Romani “patriarchs.” A gender violence court psychologist (#8) for instance described it as an archaic tradition maintained by communities who had failed to integrate into modern society:

The *Gitano* collective sometimes uses its own laws, so that [women] avoid reaching out to the justice system, and if they do, or file a complaint, they can be further endangered! That is to say, in the *Gitano* collective, there is still… (her colleague nods) one part of the collective actually has advanced, is a lot more normal and… integrated, right? […] But no, there is still a *Gitano* collective that works according to *Gitano* laws! […] And the hypothesis we can formulate is that… relationship problems of …that type… are resolved within the *Gitano* collective! They don’t resort to… gadjo^5^ law! […] So, it's an offence for the family […] for its godfather, its patriarch, who in any case imposes orders. (Me: Men of respect.) Yes, and then, she can offend them [if she] abandons the *Gitano* law and resorts to… gadjo law. And I think that those are women who can be very much endangered.

While the interviewee did clarify that she was merely “formulating a hypothesis,” she mobilized misrepresentations of Romani customs present in literature and popular culture, and to this day still shared by most of majority society, of Romani “patriarchs” ruling over families and ordering deadly punishments for women who violate community norms. In contrast, Romani social workers and mediators described the resolution process in very different terms. One mediator (#15) was especially eager to debunk the myth of “patriarchs”:

They call them patriarchs, but here we don’t have patriarchs! I am telling you, Sarah! The patriarch has always been King Juan Carlos (laughs) and currently King Felipe, and Franco! (laughs) those are persons that are patriarchal […]. Patriarchs for us don’t exist and I am saying it again. They are persons with respect. Persons who had to gain this respect over their lifetime. Persons who had knowledge and wisdom.

Known as “persons of respect” among Romani people, the elderly entrusted with resolving conflicts and emergency situations are both men and women considered wiser due to their age—not authoritarian men feared by the community. In cases of gender violence, another Romani mediator (#18) explained, “women of respect” play a primary role before “men of respect” could potentially intervene:

When a woman wants to report her husband for battery, she needs to reach out to the closest women in her family. And if those women cannot solve it among themselves, then they reach out to a *Consejo de Gitanos*. And they determine what should be done. Of course, when it's a case of gender violence, in the cases we know, they have always supported the woman. The woman keeps the house, the children, among ourselves we say that the lands are split, which means that he is the one who has to leave. If she complains about a problem she has with him, whether it is alcohol or anything else, we see that it isn’t possible to reconcile them, even if she wants to, so we gather through a *Consejo de Gitanos*. In other words, if the *gitana* woman has this recourse, she will use it before reporting to authorities! And if she wants to [report to authorities], she can! No problem! […] Elderly women, women of respect, who are older than 50, who have enough experience to know that a 25-year-old girl, who is super in love, he hit her, attacked her, she wants to leave him, perfect! But she is going to get back with him one month later, so these women filter that, they guide this woman… […] and they help her too, how do you want to organise it, what is going to happen with the children, they advise her in everything. […] They don’t leave her without protection! She is supported by them, all right?

In other words, Romani women approach community elders’ resolution as a first layer of protection they can choose to turn to before considering filing an official complaint with public authorities. It is not understood as a mandatory mechanism but, rather, as a support network available to shield them both from the perpetrator and from secondary victimization in the criminal justice system. As another Romani social worker (interviewee #19) insisted, community resolution was developed as a survival mechanism due to the hostile environments in which Romani people found themselves and has been maintained in Spain because the conventional legal system continues to represent a risk to them:

Public institutions, throughout the centuries, haven’t supported us! […] What they were doing was persecute us! What they were doing was incarcerate us! I am talking about Spain, but this concerns Europe as a whole. If you see that when you need legal support outside of your group, this structure further attacks you and creates more problems, you’re not going to resort to it! So what happens? You have to create your own internal system for defence and conflict resolution. Which already existed because we have always been an independent people. We went from country to country and we had this internal structure responding to our needs, basic needs like food, anything. When it didn’t work and you wanted to use another system, you saw it wasn’t helping you. So even more so, we have to create internal systems. So they shouldn’t complain now because we have our own internal systems, because they’re the ones who provoked it! What's going on is these systems have survived, and they are useful for many things! Why do we have to go to a slow, gadjo justice system that doesn’t consider our needs, when we have our own system for conflict resolution? […] It helps me, and I use it! But I use the means that benefit me. When it doesn’t help, you have to go somewhere else. But what happens is, in the 21st century, this external structure does not help either, because it doesn’t take our system, our values and our needs into consideration.

Importantly, she noted that “[she used] it, but [she used] the means that [benefitted her]. When it doesn’t help, you have to go somewhere else.” And indeed, all the Romani women I interviewed who shared their knowledge and experiences of community elders’ resolution insisted on the diversity of paths which Romani survivors may take. Likewise, all throughout my participant observation with them, Romani women would repeatedly remind me, “there is a bit of everything.” Even when it is shaped by 600 years of white supremacy, everyone's experience is heterogeneous, hybrid, and distinct. In that sense, what they did was defend a survivor-centered perspective, that honors the agency of every individual and leaves options for them to choose which route they consider the safest.

#### Towards TJ

While this form of resolution process can naturally also be used to enforce heteropatriarchal expectations, elements of it bear resemblance with the concept of TJ developed in the past two decades by abolitionist feminists in the USA, notably as a response to the risks faced by gender violence survivors from historically oppressed minorities when they interact with the criminal justice system. Some suggest that TJ was inspired by Restorative Justice models, attributed to 1970s Christian movements in the USA (Fernández, 2010), but also to other community practices such as those of autochtonous peoples in Canada (Smith, 2005), which prioritize reconciliation and community healing over isolating punishment mechanisms (Morris & Young, 2001). Others, on the contrary, would argue that Restorative Justice is a reformist movement that works hand in hand with criminal prosecution (Fernández, 2010) and is white-dominated, whereas TJ was developed as a movement outside and against criminal justice by historically oppressed minorities who cannot rely on police intervention for their safety (Brazzell and Meiners, in Bierria, Caruthers & Lober, 2022). Even though TJ resolution processes similarly aim at community healing and education, they explicitly embed the harm caused within broader power structures, rather than superficially looking to repair it. They are first and foremost designed from the perspective of the survivor to minimise risks of secondary victimization, giving absolute priority to their personal safety choices in a long-term commitment involving the whole community (INCITE!, 2003; Smith, 2005; Ricordeau, 2019).

Against common assumptions among Restorative Justice scholars and practitioners that TJ initiatives have worked as “feminist and queer […] late-stage add-ons” to “masculinist anti-carceral ideologies” (Bierria et al., 2022, p. 3), abolitionist feminists understand TJ as a praxis rather than a theory, rooted in the everyday strategies deployed by historically oppressed minorities to protect themselves and their loved ones in a climate of social and institutional hostility. As abolitionist scholar-activist Ruthie Gilmore has been famously reiterating, abolition is not absence, it is “radical presence” (quoted in Bierria et al., 2022, p. 2). TJ encompasses a variety of local community initiatives which all rely on the core principles of survivor-centered safety, community accountability, and social transformation, but might not bear the same name and are more centered on the survival of persecuted communities than on anti-prison advocacy per se. Inspired by Mimi Kim's 2004 Creative Interventions project in Oakland, documented TJ initiatives outside the U.S. mostly center on community responses to gender and sexual violence and seem to remain confined to English-speaking radical activist spaces experimenting with new political imaginaries (e.g., “What Really Makes Us Safe?” project, Transformative Justice Collective in Germany; Alternative Justice in India; Sisters Uncut in the UK (Brazzell and Meiners, in Bierria et al., 2022; Davis et al., 2022). However, abolitionist feminist scholars acknowledge that the attention to such projects is biased by the use of English language and the Abolition and Transformative Justice labels that originated in the USA (Davis et al., 2022). In practice, the everyday survival strategies celebrated by TJ rather consist of immediate interventions to find creative and adaptable strategies to exit interpersonal violence off the radar of policy and media attention, rather than advocacy campaigns.

As I showed, interviewed Romani women's descriptions of gender violence resolution processes through the intervention of community elders not only contrasted with the hegemonic understanding of criminal justice as the only possible route to safety. They also disproved practitioners’ representations of the so-called *ley gitana* as a rigid patriarchal system to which Romani women would have to reluctantly submit regardless of their personal needs. On the contrary, they highlighted community resolution's adaptability to survivors’ circumstances and the remodeling of usual channels in cases of gender violence. Romani women in Spain thus tend to rely on female-led community support networks when experiencing violence from their current or former partners. Elderly men are usually not called in unless the situation escalates and the whole community needs to be involved to expel the perpetrator and ensure he does not return. “Community accountability,” a core TJ principle coined by the collective INCITE! Women, Gender Non-Confirming, and Trans People of Color Against Violence (INCITE!, 2003), is in theory at least, central to Romani community interventions in cases of gender violence. Yet, as Margareta Matache importantly argues, the “mythmaking” of Romani criminality in European criminal legal frameworks was not caused by individual prejudice—it has been serving the purpose of maintaining white supremacy (Matache, 2026). In other words, the fantasies of primitive community justice which were so salient in my interviews with gender violence practitioners likely will subsist, no matter how often one might try to debunk them. What might reduce harm, however, is a better centering of Romani perspectives in the growing anti-punitive organizing for Transformative Justice among feminist activists in the region.

## Conclusion

The LOVG was a risky compromise between two opposing approaches: one that is survivor-centered and invests into material and psychosocial support on the one hand, and one aimed at stigmatizing violent men through sanction and isolation, on the other. For contenders of the former, the 2004 legal reform was hoped to introduce a shift of perspective in state intervention, from punishing deviant behavior to honoring the agency and self-formulated needs of survivors. It was expected to bring about transformation in society through collaboration with various policy areas and social actors to deconstruct destructive masculinity—and it might have, to some limited extent. Nevertheless, major reforms achieved through wide alliances unavoidably lead to conflictual agendas and interpretations of legal provisions—and, to the disappointment of many feminist advocates, the punitive dimension of state intervention ended up largely taking over. The State Pact against Gender Violence, adopted in 2017 to ensure national policies complied with the Council of Europe Convention on Preventing and Combating Violence against Women and Domestic Violence, did little to address the risks which criminal response to violence poses to historically oppressed minorities.

Admittedly, in a system where criminalization has become commonsensical, relying on legal reformism might be a lost battle. Such is the stance that the transnational abolitionist feminist movement has been defending, channeling efforts into establishing safe support networks outside of the criminal justice system instead. Nevertheless, my data analysis suggests that some organizing is also needed to undo the coercive approach defended by the LOVG and the broader legal framework. Mandatory reporting, mandatory testifying, and the obligation to proceed with a case even after charges have been dropped do not protect survivors as commonly assumed by gender violence practitioners. On the contrary, it exposes impoverished Romani women, who already are the daily target of a dense state surveillance system, to further harm. Most refuse to report acts of violence they or their peers are experiencing to state authorities, because they are aware of the tangible risks they run when doing so: financial costs, house eviction, custody withdrawal, incarceration, and retaliation in the case that they will not be offered adequate protection. Even though elderly community resolution or possibly other Transformative Justice channels exist outside the state, a legal system that coerces survivors into reporting and testifying remains a source of danger. Regardless of their chosen safety-seeking strategies should they experience gender or sexual violence, the Romani women who participated in my research were unanimous in that their agency should be respected.

Two promising developments lead us to believe that more efforts might be deployed to support Romani survivors’ help-seeking strategies in the future. One top-down initiative was launched at the Spanish Parliament (*Congreso de Diputados*) in 2021, through the creation of a Romani MPs-led subparliamentary committee against Antigypsyism, to recognize and repair the harm which the Spanish state has caused to Romani people. Arguably, the Spanish state's moves towards recognition have mostly been lip-service so far, and Romani public voices have been bitterly mocking the announcement of 2025 as the Year of the Romani People, which reportedly came without an allocated budget (Agüero Fernández, 2025)—but the institutional visibility given to Romani people is so rare in Europe that it remains worth mentioning. Simultaneously, a bottom-up movement, led by Romani feminist scholars and activists (e.g., Agüero Fernández, 2022; Asociación Gitanas Feministas por la Diversidad, 2017; Caro Maya, 2019; Filigrana, 2020, 2021), has been prolific to publicize more widely what white feminists can learn from Romani women's forms of organizing. In its 2023 manifesto, the Comisión 8M explicitly described today's feminist mobilizations as a legacy of Romani women's resistance under the Castilian regime:

We are a feminist tornado that comes from the past, that originates in the struggles of our ancestors. The tornado that brings us here today comes with the memory of Latina women, Filipina women, Rifeña women, Moroccan women, Sahrawi women, and Guinean women who faced Spanish colonialism; of women repressed by Francoism; of women harmed by his regime; of women forcefully disappeared, exiled, imprisoned, silenced, forgotten, of women we never stopped searching for. Of Romani women marginalised and despised by a Spain that defined itself as imperial, white, and Catholic. We demand for each and every one of them that the truth be brought to light, that justice be made and that the harm caused be repaired.^6^

Rather than simply discuss the specificities of Romani women's experiences of violence, we may want to consider what anti-Romani racism in gender violence institutions tells us about Spain's criminal justice system in general—instead of relegating Romani perspectives to a “footnote” in gender violence advocacy work, as Romani feminist scholar Alexandra Oprea (2012) already warned us over a decade ago.

## Appendix. List of Individual Interviewees (anonymized).

**Table:**

1	Coordinator of the Observatory for Gender Violence of the General Council of Judicial Power
2	Gender violence court judge (instruction hearings)
3	Gender violence court judge (instruction hearings)
4	Gender violence court judge (instruction hearings)
5	Gender violence court social worker (forensic reports)
6	Gender violence court social worker (forensic reports)
7	Gender violence court social worker (forensic reports)
8	Gender violence court psychologist (forensic reports)
9	Gender violence court judicial secretary
10	Gender violence specialized criminal judge (criminal hearings)
11	Gender violence specialized Magistrate at the Superior Tribunal of Justice
12	Local police special unit for women, youth, and elderly
13	Local police special unit for women, youth, and elderly
14	Local police special unit for women, youth, and elderly
15	Romani community mediator
16	Romani community mediator
17	Romani community mediator
18	Romani community mediator
19	Romani feminist activist
20	Romani feminist activist
21	Romani feminist activist
22	Romani feminist activist
23	Romani feminist activist

## Notes

1. Translated by the author.

2. *Gitano* (masculine form) or *Gitana* (feminine form) literally translates into “Gypsy.” Unlike “Gypsy,” which is now widely recognized as a racial slur historically imposed by majority society on Romani people and other groups, *Gitano/a* has been reappropriated by the local Romani minority. However, it still bears strong racial connotations and remains frequently used as an insult in Spain.

3. “Acuerdo del 7 de enero de 2025, por el que se aprueba la declaración institucional con motivo del 600° aniversario de la llegada del pueblo gitano a España y se declara el año 2025 como Año del Pueblo Gitano en España,” https://www.dsca.gob.es/es/600-anos-pueblo-gitano/declaracion-2025

4. Here referred to as gender violence specialized courts, for ease of clarity.

5. Gadjo means white non-Romani in Romani language. The more commonly used word in Spain is *payo*. The expression the interviewee uses in the original is *la ley paya*.

6. Translated by the author.
